# Functional characterization of the native swollenin from *Trichoderma reesei*: study of its possible role as C_1_ factor of enzymatic lignocellulose conversion

**DOI:** 10.1186/s13068-016-0590-2

**Published:** 2016-08-26

**Authors:** Manuel Eibinger, Karin Sigl, Jürgen Sattelkow, Thomas Ganner, Jonas Ramoni, Bernhard Seiboth, Harald Plank, Bernd Nidetzky

**Affiliations:** 1 Institute of Biotechnology and Biochemical Engineering, Graz University of Technology, Petersgasse 12/1, 8010 Graz, Austria; 2 Institute for Electron Microscopy and Nanoanalysis, Graz University of Technology, Steyrergasse 17, 8010 Graz, Austria; 3 Research Division Biochemical Technology, Institute of Chemical Engineering, TU Wien, Gumpendorferstrasse 1A/166, 1060 Vienna, Austria; 4 Graz Centre for Electron Microscopy, Steyrergasse 17, 8010 Graz, Austria; 5 Austrian Centre of Industrial Biotechnology, Petersgasse 14, 8010 Graz, Austria

**Keywords:** SWO1, Swollenin, Expansin, *Trichoderma reesei*, Glycoprotein, Cellulose degradation, Synergism, Amorphogenesis, Atomic force microscopy

## Abstract

**Background:**

Through binding to cellulose, expansin-like proteins are thought to loosen the structural order of crystalline surface material, thus making it more accessible for degradation by hydrolytic enzymes. Swollenin SWO1 is the major expansin-like protein from the fungus *Trichoderma reesei*. Here, we have performed a detailed characterization of a recombinant native form of SWO1 with respect to its possible auxiliary role in the enzymatic saccharification of lignocellulosic substrates.

**Results:**

The *swo1* gene was overexpressed in *T. reesei* QM9414 Δ*xyr1* mutant, featuring downregulated cellulase production, and the protein was purified from culture supernatant. SWO1 was *N*-glycosylated and its circular dichroism spectrum suggested a folded protein. Adsorption isotherms (25 °C, pH 5.0, 1.0 mg substrate/mL) revealed SWO1 to be 120- and 20-fold more specific for binding to birchwood xylan and kraft lignin, respectively, than for binding to Avicel PH-101. The SWO1 binding capacity on lignin (25 µmol/g) exceeded 12-fold that on Avicel PH-101 (2.1 µmol/g). On xylan, not only the binding capacity (22 µmol/g) but also the affinity of SWO1 (*K*_d_ = 0.08 µM) was enhanced compared to Avicel PH-101 (*K*_d_ = 0.89 µM). SWO1 caused rapid release of a tiny amount of reducing sugars (<1 % of total) from different substrates (Avicel PH-101, nanocrystalline cellulose, steam-pretreated wheat straw, barley β-glucan, cellotetraose) but did not promote continued saccharification. Atomic force microscopy revealed that amorphous cellulose films were not affected by SWO1. Also with AFM, binding of SWO1 to cellulose nanocrystallites was demonstrated at the single-molecule level, but adsorption did not affect this cellulose. SWO1 exhibited no synergy with *T. reesei* cellulases in the hydrolysis of the different celluloses. However, SWO1 boosted slightly (1.5-fold) the reducing sugar release from a native grass substrate.

**Conclusions:**

SWO1 is a strongly glycosylated protein, which has implications for producing it in heterologous hosts. Although SWO1 binds to crystalline cellulose, its adsorption to xylan is much stronger. SWO1 is not an auxiliary factor of the enzymatic degradation of a variety of cellulosic substrates. Effect of SWO1 on sugar release from intact plant cell walls might be exploitable with certain (e.g., mildly pretreated) lignocellulosic feedstocks.

## Background

Through integrated developments in pretreatment technologies and cellulase engineering, much progress has been made in enhancing the efficiency of soluble sugar release from lignocellulosic feedstocks [1, 2]. However, the enzyme costs incurred in the saccharification step are still significant [2, 3]. There is high interest, therefore, in further decreasing the enzyme loading required in the process. Besides making the cellulases more effective *per se*, through improving their intrinsic activity [4–6] and facilitating their production [5, 7, 8], the reinforcement of existing cellulase preparations by auxiliary proteins and enzymes has attracted considerable attention [5, 9–13]. Lytic polysaccharide monooxygenase is a prominent example of an auxiliary enzyme, which is already in use to supplement cellulase preparations [10, 11, 14, 15]. Proteins lacking enzyme activity could also, in different ways, exert an auxiliary function in cellulose bioconversion. Inspired by Elwyn Reese’s early C_1_–C_x_ postulate (or updated variants thereof), invoking a non-hydrolytic, cellulose structure-disrupting C_1_ factor that acts in synergy with hydrolytic enzymes (the C_x_ factor), the discovery of a possible C_1_ factor of cellulose degradation has been a clear focus of interest in the research on auxiliary proteins [5, 10, 16].

Originally discovered from plants as cell wall-loosening proteins, expansins and expansin-like proteins constitute a widely distributed superfamily of proteins [17–19]. Besides plants, phylogenetically diverse microorganisms including bacteria and fungi, most of which grow in association with plants, were also found to contain expansins [20, 21]. Biologically, expansins are described to function as physical catalysts of cell wall enlargement and stress relaxation in plants. They appear to do so by promoting a rearrangement in the network of non-covalent interactions between the cell wall polysaccharides, in particular, those matrix glycans that interconnect individual cellulose microfibrils [19, 22]. By partly disrupting the bonding these glycans have to the microfibril surface and to each other, expansin action is supposed to enable the displacement of the cell wall polymers and, thus, to promote slippage in the points of their adhesion [19, 22, 23]. In microorganisms, expansins appear to be important factors of the colonization of plant tissues [24–27]. Although expansins do not weaken the cell wall or cause a lasting change in the wall structure [19] (except altering its size and shape [28]), they might, however, cause processes, sometimes referred to collectively as “amorphogenesis”, in which cellulose or lignocellulose structures become disaggregated and loosened up. This amorphogenesis, and the beneficial effect it might have on the action of hydrolytic enzymes could make expansins broadly useful in cellulosic biomass conversion [21, 29–31].

Originally discovered by Saloheimo and colleagues [25] who showed it to cause swelling of cotton fibers, swollenin is a special expansin-like protein from fungi. It differs from the canonical expansins in size (~493 compared to ~225 amino acids) and also in the arrangement of structural modules within the protein structure. Expansins are modular proteins built of two discrete domains connected by a short linker [5, 26, 32]. The N-terminal domain shows weak resemblance to the catalytic module of family GH-45 glycoside hydrolases, lacking their full catalytic machinery, however [5, 25]. We refer to this aspect later under “Results” section, but expansins are generally described to lack polysaccharide hydrolase activity. The C-terminal domain resembles certain carbohydrate-binding modules (e.g., CBM family 3 or 63) [25, 33]. Both domains are required for the full cell wall-loosening activity of the expansins [32, 33].

Swollenin deviates from the basic expansin conformation by having an additional CBM from family 1 located N-terminally [5, 18, 25]. The expansin-like domain and the family 1 CBM are connected by a putative linker and/or fibronectin-III (Fn-III)-like domain [18, 34]. Linkers, in general, serve as flexible elements in protein structures [18, 35, 36]; however, little is currently known about the actual role of the linker region in swollenin. It is noted though that multiple Ser/Thr residues for *O*-glycosylation are present in the linker/Fn-III-like domain of swollenin [5, 37].

Expansin/swollenin “activity” has been assayed in different ways but it is generally difficult to evaluate. A biomechanical assay measures directly the effect of the protein on the fiber strength of the cellulosic material [19, 32]. Light microscopy was used often to track fiber disaggregation and other morphological changes in cellulosic material on incubation with swollenin [18, 34, 37–39]. Cellulose crystallinity was also determined to monitor the amorphogenesis [37, 38, 40]. To identify and characterize swollenin-caused changes in the surface properties of cellulose, biological methods (e.g., adsorption of CBM [41, 42]) and high-resolution microscopy (SEM and AFM) were used [18, 37, 41]. Synergy with cellulases in releasing soluble sugars from lignocellulosic substrates presents a highly indirect but use-inspired way of expressing swollenin activity [34, 37, 40, 43–45]. Table 1 summarizes the results from different papers analyzing the possible involvement of swollenin in the degradation of lignocellulosic substrates. The studies were selected because besides synergy with cellulases, which has been the topic of numerous papers, they also examined the effect of swollenin on the morphology of the cellulosic substrate used. As it becomes clear from Table 1, the current literature does not offer a conclusive picture, thus motivating the present study to obtain clarification.

**Table 1:** Summary of reported structural changes in lignocellulosic substrates caused by swollenin preparations obtained through different strategies of protein expression and production

Native source/produced in/purification/aMm	Substrates	Experimental setup; employed methods	Effects	Ref.
*T. reesei*/*S. cerevisiae*/CS/~75 kDa	Mercerized cotton fibers	0.25 µg_Swo_/g_substrate_, 25 °C, 4 h; light microscopy	Local disruption of cotton fibers, no release of sugars	[18]
*T. reesei*/*S. cerevisiae*/CS/~75 kDa	Whatman No. 3 filter paper	5 mL CS/filter paper strip, room temperature, 15 min; paper strength test	Reduction of tensile strength and average peak load (15–20 %)	[18]
*T. reesei*/*A. niger*/AC/~80–95 kDa	*Valonia* sp. cell wall fragments	10 µg_Swo_/g_substrate_, 45 °C, 48 h; AFM, light microscopy	Partial disintegration to isolated fibers, no release of sugars	[18]
*A. fumigatus*/*A. oryzae*/AC/~85 kDa	Avicel PH-101, filter paper (603 cellulose thimbles)	0.8 µg_Swo_/mg_Avicel_, 8 µg_Swo_/mg_filter paper_, 40 °C, 72 h; light microscopy, visual examination	Avicel PH-101 particle size reduction (~50 %), effect is pH- and temperature-dependent; complete disruption of filter paper, no release of sugars	[34]
*T. reesei*/*K. lactis/*IMAC/~100 kDa	Whatman No. 1 filter paper, α-cellulose, Avicel PH-101, sigmacell 101	20 µg_Swo_/mg_substrate_, 45 °C, 48 h; XRD, laser diffraction	Reduction of CrI (~10 up to 22 %) and particle size (up to ~30 %) was observed for all substrates except Sigmacell	[37]
*T. reesei*/*K. lactis*/IMAC/~100 kDa	Whatman No. 1 filter paper	20 µg_Swo_/mg_substrate_, 45 °C, 48 h; SEM, photography	Deagglomeration of filter paper (reduction of particle size and count); SEM showed an increased surface roughness; no swelling was observed	[37]
*T. asperellum*/*E. coli*/refolding, AC/~35–50 kDa	Avicel PH-101	5 µg_Swo_/mg_substrate_, 50 °C, 91 h; light microscopy	Partial disruption of Avicel PH-101 particles	[38]
*T. pseudokoningii*/*A. niger*/HIC/~75 kDa	Avicel PH-101, filter paper	5–20 µg_Swo_/mg_Avicel_, 0.5–2 µg_Swo_/mg_filter paper_, 40 °C, 48–72 h; light microscopy, XRD	No effects were observed by applying light microscopy; CrI was increased (88–90 %)	[40]
*T. reesei*/*T. reesei*/IMAC, IE/n.a.	Mercerized cotton fibers	10 µg_Swo_/mg_substrate_, 50 °C, overnight; CBM adsorption assay, SEM	Available surface for CBMs was increased (~38 %); SEM showed a smoothed surface upon *Tr*Swo1 treatment	[41]
*T. reesei*/*E. coli* and *N. tabacum*/CS/n.a.	Mercerized cotton fibers	0.2–2 µg_Swo_/mg_substrate_, 37 °C or 50 °C, 8 h; phase contrast microscopy	Fiber expansion, inner fiber structure was altered independent of the *Tr*Swo1 source	[28]
*P. oxalicum*/*T. reesei*/precipitation, IMAC/~90 kDa	Avicel PH-101	4 µg_Swo_/mg_substrate_, 50 °C, 48 h; light microscopy, protein binding assay	Partial disruption of Avicel PH-101 particles; *B*_max_ for cellulases was increased (~20 %)	[39]
*T. reesei*/*T. reesei*/IMAC, IE/n.a.	Dissolving pulp, various lignocellulosic pulps	50 µg_Swo_/mg_substrate_, 50 °C, overnight; high-resolution fiber quality analyzer	Fragmentation was observed to a low extent for dissolving pulp fibers but not for lignocellulosic pulps	[42]
*Orpinomyces* sp. strain C1A/*E. coli*/refolding, IMAC/~67 kDa	Cotton fibers	0.25–5 µg_Swo_/mg_substrate_, 39 °C, 12 h; ESEM, Congo red cotton assay	Average cotton fiber width was increased (~56 %); dye adsorption was increased (CAE ~0.4 for 5 µg_Swo_/mg_substrate_)	[78]

*aMm* apparent molecular mass, *CS* enriched culture supernatant, *AC* affinity chromatography, *IMAC* immobilized metal adsorption chromatography (via His-tag), *HIC* hydrophobic interaction chromatography, *IE* ion exchange chromatography, *n.a.* not available

In the absence of a clear parameter able to capture the functionality of swollenin in an alleged amorphogenesis process, it is crucially important in the search of cause-and-effect relationships that the protein preparation used is well defined in its main structural characteristics. In addition, for the purpose of rigorously establishing an intrinsic biological reference, the swollenin should be as native-like as possible. Previous studies have obtained the recombinant swollenin through heterologous expression of the coding gene in foreign hosts (e.g., *Escherichia coli*, *Pichia pastoris*, *Saccharomyces cerevisiae*, *Aspergillus* sp.) [18, 28, 37–40, 43, 45], bearing in each case the risk that non-native post-translational processing, especially glycosylation, could affect the protein function [46, 47]. Fusion tags were also used to facilitate protein purification [42, 44, 48], but whether these modifications of the native structure are functionally silent or interfere with the original function of the swollenin is not known. The yield of recombinant swollenin from heterologous production was usually in the low (≤20–100) mg/L culture range [21], and to our knowledge, proper folding of the recombinant protein was never assessed.

In this study of SWO1, the major swollenin of *Trichoderma reesei* (anamorph *Hypocrea jecorina*), we addressed the urgent concern about the nativeness of recombinant swollenin by producing the protein via homologous overexpression in the native host. To largely eliminate the otherwise huge cellulase and hemicellulase background in the secretome of *T. reesei*, a mutant QM9414 strain was used in which, as shown in earlier work, the hydrolytic enzyme production was strongly downregulated due to *xyr1* transcriptional regulator gene knockout [49]. Using the target protein thus produced and purified from culture supernatant, a detailed functional characterization of SWO1 was performed. Besides adsorption studies, this included direct measurements of a possible cellulose structure-disrupting activity of SWO1. Synergy with *T. reesei* cellulases was evaluated during degradation of different cellulose substrates.

## Results

### Recombinant production and purification of the native SWO1

**Fig. 1:**
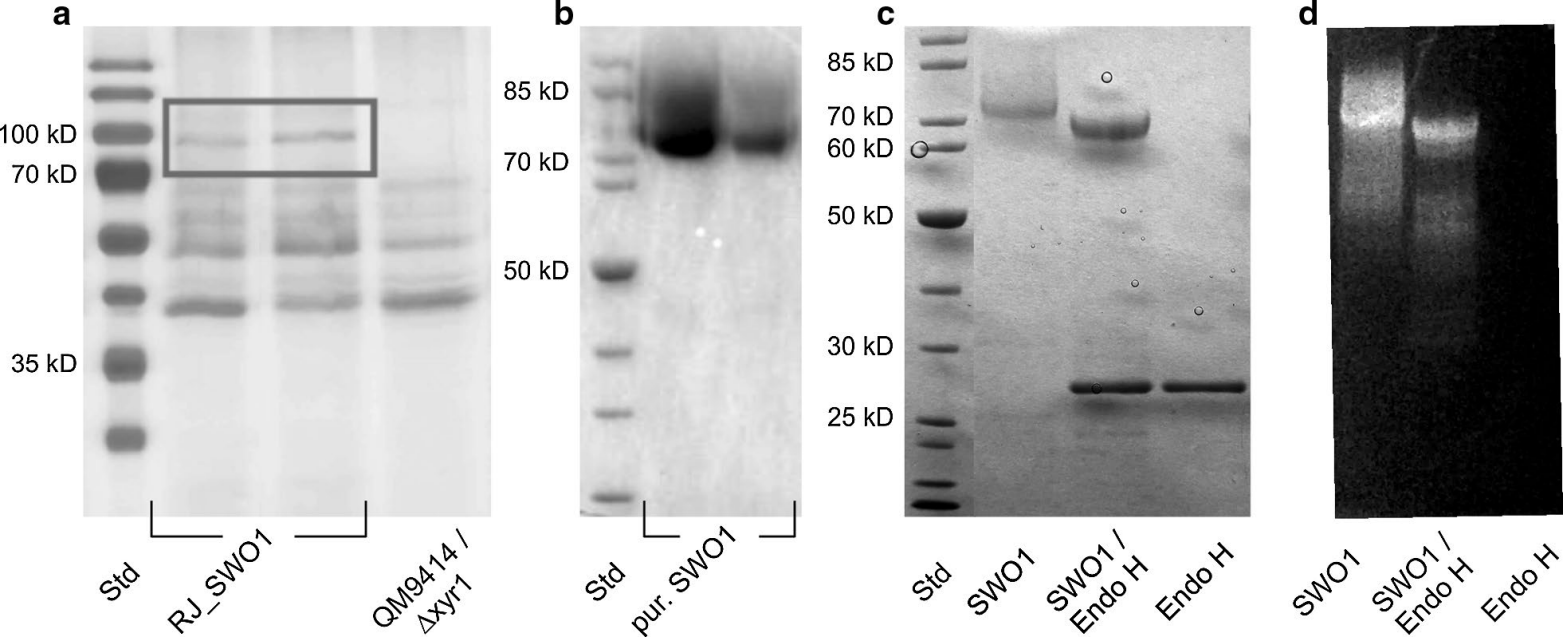
Identification, purification and deglycosylation of SWO1. **a** SWO1 (indicated with a *rectangle*) was recombinantly expressed in *T. reesei* QM9414 ∆xyr1 (designated as RJ_SWO1) and secreted into the culture media (two independent fermentations are shown). A prominent band at about 75 kDa, which was absent in an untransformed control strain, was identified as SWO1. **b** Single-step batch chromatography on Avicel PH-101 as adsorbent was used to purify SWO1 (two independent purifications are shown). The purified protein migrated as a single but relatively diffuse protein band, suggesting that the recombinant SWO1 was strongly glycosylated. **c** Deglycosylation of SWO1 with Endo H resulted in a decrease of the apparent molecular mass by roughly 5 kDa and a more sharply focused protein band was obtained in the SDS-polyacrylamide gel. **d** Direct glycostaining of the same gel shown in **c** confirmed the presence of glycans on both the native and the Endo H-treated SWO1

The genetic background of *T. reesei* QM9414 Δ*xyr1* was previously shown to present a useful vehicle for the overexpression of individual secreted proteins in an overall cellulase- and hemicellulase-free environment [49]. The coding region of the *swo1* gene was, therefore, integrated genomically under control of the *cdna1* promoter. In positive recipient strains, the presence of the *swo1* expression cassette was verified by PCR, and secretion of the target protein into the culture supernatant was also clearly indicated in SDS-PAGE. The recombinant SWO1 was produced in a 2-L bioreactor cultivation of the *T. reesei* integrant strain, using glucose as the carbon source. The fungal secretome comprised SWO1 as a major protein component, as shown in Fig. 1a. Prominent protein band at about 75 kDa mass which was absent from the supernatant of the control strain indicated secretion of recombinant protein. Using batch chromatography on Avicel PH-101 as an affinity adsorbent, SWO1 was isolated from the supernatant in just a single step of downstream processing, as shown in Fig. 1b. The protein yield was about 4 mg/L of culture. The apparent molecular mass of SWO1 in SDS-PAGE (~75 kDa) differed substantially from the mass of 49 kDa expected from protein’s amino acid sequence, consistent with the observations of Saloheimo et al. [18] who used Western blotting for detection of SWO1 in *T. reesei* culture supernatants. This unusually high molecular mass plus the fact that the purified protein migrated as a single but relatively diffuse band in SDS-PAGE suggested that the recombinant SWO1 was strongly glycosylated. There are four *N*-glycosylation sites in the sequence of SWO1, three of which are identified as strong candidates to become glycosylated [48]. The linker/Fn-III-like domain might be additionally *O*-glycosylated [18, 37, 48]. Figure 1c shows that on incubation with Endo H for removal of protein *N*-glycans, the apparent molecular mass of the native SWO1 was only slightly reduced (~5 kDa). This suggests that the difference between the calculated and observed molecular mass cannot be explained only as a result of *N*-glycosylation, and that *O*-glycosylation might contribute to the difference. Direct glycostaining in the gel confirmed the presence of glycans on both the native and the Endo H-treated SWO1 (Fig. 1d). We, therefore, concluded that the recombinant SWO1 was *N*-glycosylated but also strongly *O*-glycosylated.

### Structural analysis of SWO1 using circular dichroism spectroscopy and protein modeling

**Fig. 2:**
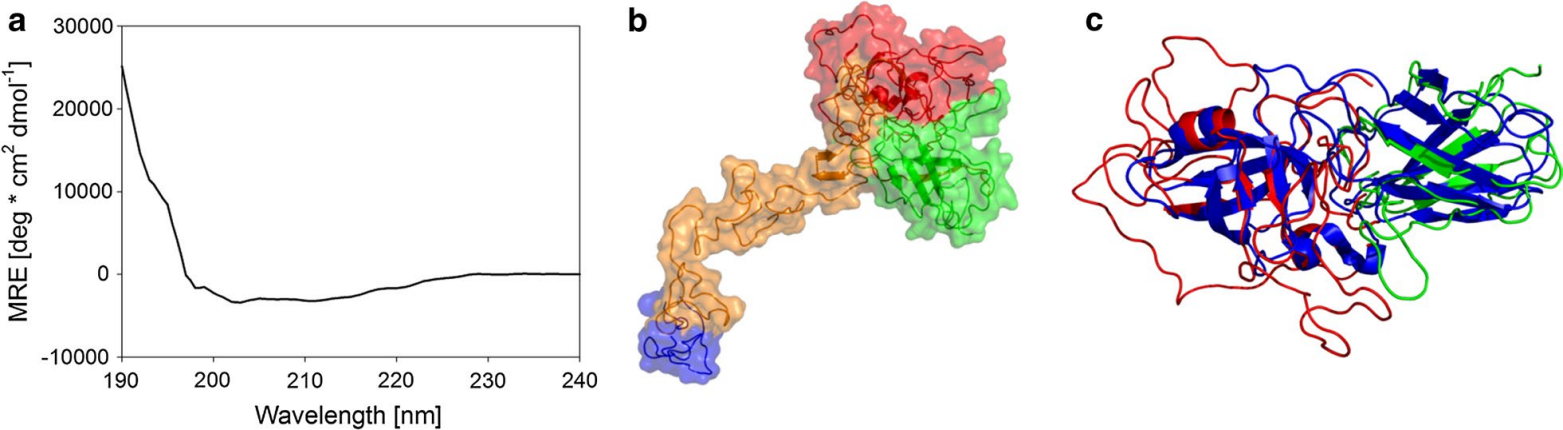
CD spectra and homology model of native SWO1. **a** Smoothed CD spectrum of native SWO1 in 50 mM sodium acetate buffer, pH 5.0, at room temperature. For further details of the measurement, see the “Methods” section. **b** The automated protein structure homology-modeling server Phyre2 was used to predict the protein structure of SWO1 (UniProt ID: Q9P8D0). The distinct domains are colored as follows: family 1 CBM (*blue*); linker/Fn-III-like domain (*orange*); GH45 domain (*red*); expansin-like CBM (*green*). **c** A structural overlay of the modeled expansin-like domain from SWO1 with EXLX1 from *B. subtilis* (PDB ID: 4FG2) was made to allow visual comparison. The distinct domains are colored as follows: EXLX1 (*blue*); GH45 domain (*red*); expansin-like CBM (*green*). Overall, 87 % of the input sequence was modeled at >90 % confidence, and 61 residues were modeled ab initio. The depicted model was used to calculate the percentage of secondary structure elements

The far-UV CD spectrum of purified SWO1 is shown in Fig. 2a. This suggested a folded protein with a high content of β-strand relative to α-helical secondary structure. It also indicated a large portion of the protein structure to lack a discrete organization into secondary structural elements. Analysis of the spectrum with DichroWeb suggested SWO1 to be composed of just 7 % α-helices, 34 % β-strands, 18 % turns and 34 % unordered structure. Structural modeling of SWO1 (UniProt ID: Q9P8D0) was done with Phyre2 [50], analyzing the entire protein except for the amino acids 1–18, which are predicted to be cleaved off after secretion. Figure 2b shows a hypothetical structure of SWO1 rendered from the models of the two modules. Due to the amount of proteins with known three-dimensional structure (*N* ≥ 10) resembling either expansins or CBMs and sufficient sequence similarity, the structural models of the family 1 CBM (CBD form EG I, sequence identity: 50 %) and the expansin-like domain (e.g. β-expansin from maize, sequence identity: 28 %) appear to be plausible. In addition, a structural overlay of the modeled expansin-like domain with a crystallized bacterial expansin (EXLX1 from *B. subtilis,* PDB ID: 4FG2) is provided in Fig. 2c to allow a visual comparison of the related domains. Overall, 87 % of the input sequence was modeled at >90 % confidence, and 61 residues were modeled ab initio. Moreover, the relative content of secondary structure elements calculated from the structure model was reasonably similar to that determined from the CD spectrum. It was 3 % α-helices, 23 % β-strands, 41 % turns and 33 % unordered structure. Finally, the sequence-based prediction tool (JPred4) [51] suggested a similar content of α-helices (3 %) and β-strands (20 %). In summary, these results provided good evidence suggesting that the SWO1 as isolated was most likely properly folded.

### Adsorption of SWO1 to insoluble polysaccharides

**Fig. 3:**
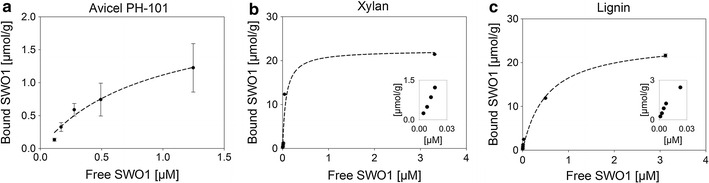
Adsorption isotherms of purified native SWO1 on lignocellulose components. Experiments were done at 25 °C in 50 mM sodium acetate, pH 5.0, over 2 h with shaking (500 rpm). Substrate concentration was 1 mg/mL in a total reaction volume of 200 µL. *Symbols* show the measured data and *error bars* show the SD from three independent experiments. *Insets* present a zoomed view on the initial data points for SWO1 adsorption on xylan and kraft lignin. The fitted Langmuir isotherms are shown as *dashed lines* and the corresponding parameters, maximum binding capacity related to the unit mass of substrate (*B*_max_) and the dissociation constant *K*_d_, are summarized in Table 2

Isotherms for the adsorption of SWO1 to Avicel PH-101, birchwood xylan and kraft lignin were determined at 25 °C and pH 5.0 employing the insoluble substrate in a concentration of 1.0 mg/mL. Using Avicel PH-101 under comparable conditions, preliminary experiments showed that most of the SWO1 was bound rapidly within 30 min and that apparent adsorption equilibrium was reached after 60 min. In accordance with an earlier study from Jäger et al. [37], isotherms were, therefore, obtained from incubations for 120 min, and the results are shown in Fig. 3. With each substrate, the binding of SWO1 appeared to correspond to a simple Langmuir adsorption isotherm. Note that Avicel PH-101 contains minor fractions of xylan (<2 %) and lignin (<1 %) [52, 53]. However, nothing is known about their localization and structural organization. For this reason and also supported by the seemingly appropriate fit (*R*^2^ ≥ 0.98), we do not consider the use of an alternative multiple binding site isotherm for Avicel PH-101. The corresponding fit of the data gave the binding constants summarized in Table 2. Remarkably, the binding capacity (*B*_max_) of SWO1 was much higher (≥12-fold) on xylan and lignin than it was on Avicel PH-101. The binding affinity in terms of the reciprocal dissociation constant (*K*_d_) was also higher (11-fold) on xylan than on Avicel PH-101. In terms of *B*_max_/*K*_d_, therefore, the specificity of SWO1 for binding to xylan exceeded that for binding to Avicel PH-101 almost 120-fold. The specificity for binding to lignin lay in between the two (Table 2). Based on *K*_d_ values reported, recombinant SWO1 obtained by heterologous expression in *Kluyveromyces lactis* bound to Avicel PH-101 with similar affinity as the natively produced SWO1 does. This result could be interpreted to mean that the binding affinity of SWO1 on Avicel PH-101 is not majorly affected by the degree of nativeness its glycosylation has. Interestingly, expansin-like proteins of bacterial origin bound to Avicel PH-101 with *K*_d_ values similar to that of SWO1, suggesting perhaps that the glycosylation is not a crucial element of binding as far as the *K*_d_ is concerned. Only to note, cellulases harboring a family 1 carbohydrate-binding module showed *K*_d_ values of Avicel PH-101 binding agreeing with the *K*_d_ of SWO1 within the same order of magnitude [37, 54, 55].

**Table 2:** Summarized adsorption parameters of SWO1 on Avicel PH-101, birchwood xylan and lignin

Substrate	Avicel PH-101	Lignin	Xylan
*B*_max_ (µmol/g)	2.11 ± 0.39	25.1 ± 1.48	22.3 ± 3.13
*K*_d_ (µM)	0.89 ± 0.30	0.53 ± 0.11	0.08 ± 0.04
Absolute specificity (L/g)^a^	2.4	47.4	279
Relative specifity^b^	1.00	20.0	118

SWO1 showed the highest affinity and specificity for xylan followed by lignin and pure cellulose. *B*_max_ maximum binding capacity, *K*_d_ dissociation constant

^a^ *B*_max_/*K*_d_

^b^Absolute specificities normalized on Avicel PH-101

### Is the native SWO1 active on its own in the depolymerization of glycan substrates?

**Fig. 4:**
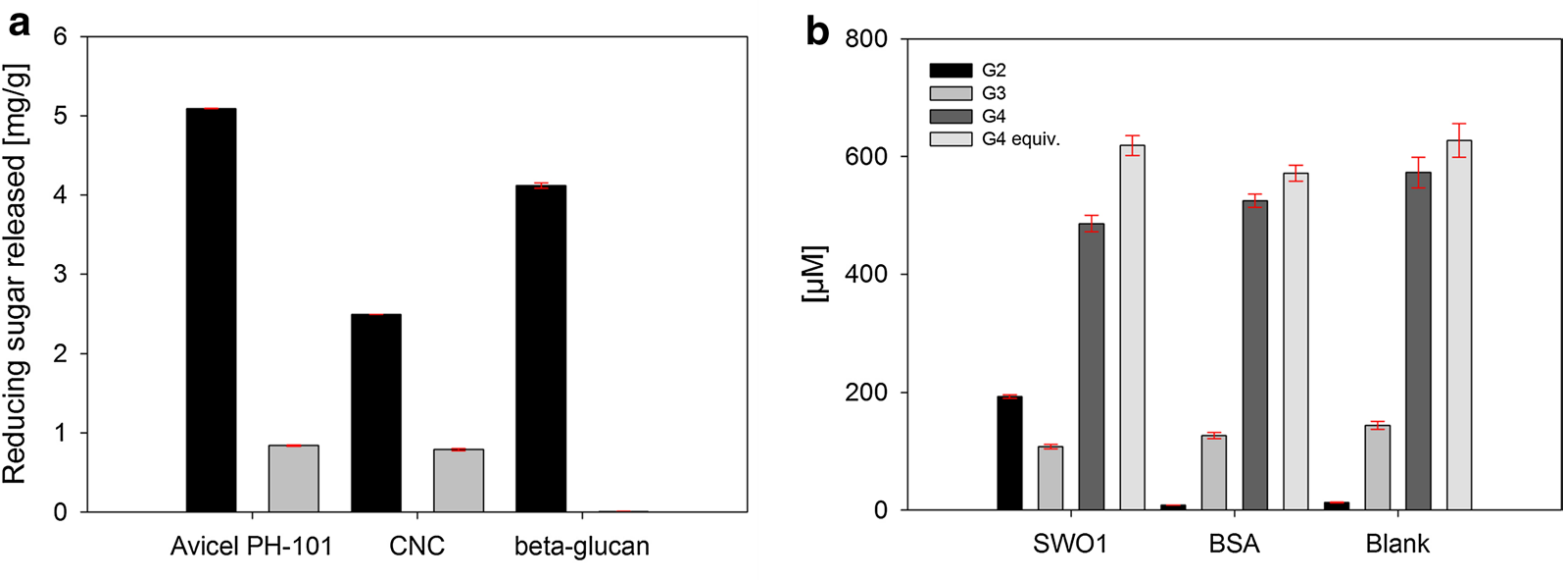
Activity of SWO1 on various glycan substrates. **a** The substrates used were Avicel PH-101, CNC and β-glucan (1 mg/mL each) in 50 mM sodium acetate buffer, pH 5.0. Incubation was for 24 h at 40 °C with shaking (500 rpm). Avicel PH-101 and CNC were incubated with 0.4 µM SWO1 (*black bars*) or an equimolar amount of BSA (*grey bars*). Reactions were stopped by heating and incubated with β-glucosidase. The glucose released was measured with an enzymatic assay. *Error bars* show SD from four independent experiments. Barley β-glucan was incubated with either 0.2 µM SWO1 or BSA. The liberated sugars were assayed with HPAEC-PAD, and cellobiose was identified as the main product of SWO1 activity. *Error bars* are from two independent experiments. **b** Cellotetraose (0.5 mg/mL) was incubated with either 0.5 µM SWO1 or BSA in 50 mM sodium acetate buffer, pH 5.0, for 24 h at 40 °C with shaking (500 rpm). The product distribution (*G2* cellobiose, *G3* cellotriose, *G4* cellotetraose) was determined with HPAEC-PAD. *Error bars* were estimated from two independent experiments

Andberg et al. [48] reported a His-tagged preparation of SWO1 to promote the release of reducing sugars from different substrates, in particular, barley β-glucan, whose conversion involved a remarkably high specific activity of SWO1 of 7 U/mg. The SWO1 produced and purified from *Aspergillus niger* var. *awamori* showed a similar specific activity on β-glucan substrate. As these findings implied SWO1 to be active enzymatically, with certain substrates at least, we also examined the native SWO1 for its ability to degrade different insoluble glycans via soluble sugar release. Figure 4a shows the results. Compared to incubations in which BSA was used as an inactive control, incubations with SWO1 caused a slightly enhanced sugar formation. However, only a tiny amount of soluble sugars was produced in comparison to the total sugar available from the substrates in polymeric form. Sugars released from cellulose were detected as glucose, because β-glucosidase was added to the reaction samples later. The product released from the β-glucan was shown to be mainly cellobiose (≥90 %). We calculated that the sugar release from the β-glucan (Fig. 4a) would correspond to a specific activity of only around 0.1 mU/mg, equivalent to just six turnovers of SWO1 within 24 h. Andberg et al. [48] observed a comparable conversion (≤1.2 mg/g) when studying the conversion of β-glucan. However, their reported specific activity of SWO1 was increased by orders of magnitude (7 U/mg). The apparent conflict in these findings is resolved by considering that the kinetics of sugar formation by SWO1 was completely unlike a “normal” enzymatic reaction. They reported a rapid accumulation of sugar in the supernatant initially (≤10 min) but no further release on prolonged incubation up to 24 h.

In our view, therefore, these effects of SWO1 appear inconsistent with its action as a true cellulase, however, weakly active. An alternative explanation is that the observable SWO1 “activity” on Avicel PH-101 and CNC resulted from the release into solution of sugars (e.g., short oligosaccharides) that were initially tightly associated with the solid material but became detached on binding of the SWO1. Note: even BSA, which we assume to interact completely unspecifically with the cellulose preparations used, caused release of trace amounts of soluble sugars as reported previously [44] (Fig. 4a).

The sugar release from barley β-glucan (Fig. 4a) might, however, reflect a tiny intrinsic hydrolase activity of SWO1. BSA does not produce detectable sugars from this substrate. Following Andberg et al., we, therefore, also examined cellotetraose as a substrate of SWO1 and show the results in Fig. 4b. While being absent from the controls, cellobiose was clearly formed in the incubation with SWO1. The amount of cellobiose released (200 µM) was explained by the cellotetraose converted (100 µM). Since no glucose was formed, the cleavage of cellotetraose appeared to have been quite specific. Utilization of the oligosaccharide substrate was also specific because cellotriose, which was present in the reaction from the beginning due to the composition of the commercial cellotetraose preparation, was not attacked at all by the SWO1. The turnover of the cellotetraose substrate by SWO1 was extremely low (0.14 min^−1^). It is called in remembrance that the C-terminal domain of SWO1 resembles structurally the catalytic modules of family GH-45 glycoside hydrolases but lacks their full active-site machinery due to the absence of a catalytic base. Therefore, considering the low level of activity that family GH-45 enzymes retain on substitution of their catalytic base by a non-functional residue [5, 26], one would not expect SWO1 to be a proficient catalyst of the hydrolysis of glycosides.

We also assayed the hydrolysis of 4-methylumbelliferyl-β-d-cellobioside, a suitable substrate for various cellulases [5, 56], but did not observe activity of the purified SWO1 in any of the possible cleavage modes, releasing 4-methylumbelliferone or 4-methylumbelliferyl-β-d-glucoside.

### Effect of SWO1 on cellulose crystallinity measured by wide-angle x-ray scattering

**Fig. 5:**
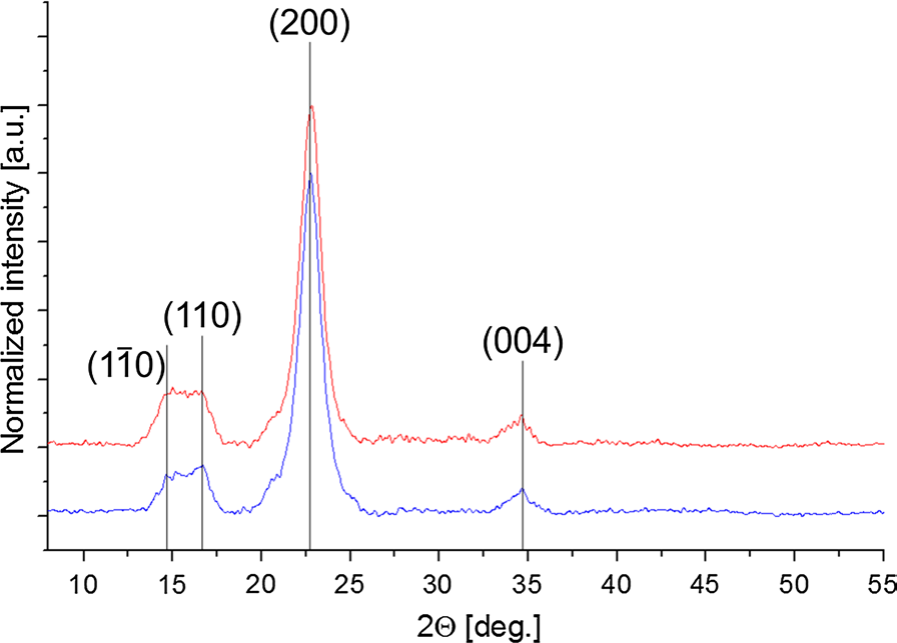
Stacked WAXS profiles of SWO1-treated and untreated Avicel PH-101. Avicel PH-101 (10 mg/mL) was incubated in 50 mM sodium acetate buffer, pH 5.0, with 0.01 µM SWO1 (*red*) or without enzyme (*blue*), for 72 h at 40 °C with agitation (150 rpm). Relevant peaks for cellulose I_β_ were resolved and indexed with Miller indices. No changes in intensity or peak’s shape and position were observed

Avicel PH-101 was incubated for 72 h in the presence of an SWO1 concentration, which according to the adsorption isotherm (Fig. 3; Table 2) was “catalytic” (0.05 mol %) relative to the available binding sites on this substrate. We considered that a possible amorphogenesis, caused by the dynamic action of SWO1, might be detectable as a decrease in the overall crystallinity of Avicel PH-101 which we measured by wide-angle x-ray scattering (WAXS) [57–59]. The results are shown in Fig. 5. A lowering of the crystallinity index would show in the WAXS profile as an intensity decrease in the major scattering peak at about 22.7°. Changes in the peak’s shape and position would also indicate transformations of the original crystalline cellulose (allomorph I_β_) into another allomorph or into amorphous material [58, 60]. Figure 5 is clear in showing that SWO1 had no effect on Avicel PH-101 structure to the extent detectable with the WAXS method used. This result contrasts, to some extent, with the findings of Jäger et al. [37] who reported changes in Avicel PH-101 crystallinity index on incubation with a *Tr*SWO1 produced recombinantly in *K. lactis*.

### Effect of SWO1 on fully amorphous and highly crystalline cellulose preparations measured by atomic force microscopy in a liquid environment

We considered that while global parameters of cellulose structural organization such as the crystallinity index (see Fig. 5) might not be suitable to capture the relevant components of an SWO1-caused amorphogenesis, a method able to reveal even subtle changes in cellulose surface morphology, and to do so in a time and laterally resolved manner, could be very useful for the identification and characterization of hidden SWO1 functions. Like in our previous studies of cellulases [57, 61, 62] and LPMO [12], atomic force microscopy (AFM) in a liquid environment was used, because the protein function could, thus, be analyzed in a setting modeled on the natural process. To contrast the effect of two completely different types of cellulose, we examined a fully amorphous cellulose film (ATFC) and cellulose nanocrystals (CNC), both spin-cast on silicon wafers [57].

**Fig. 6:**
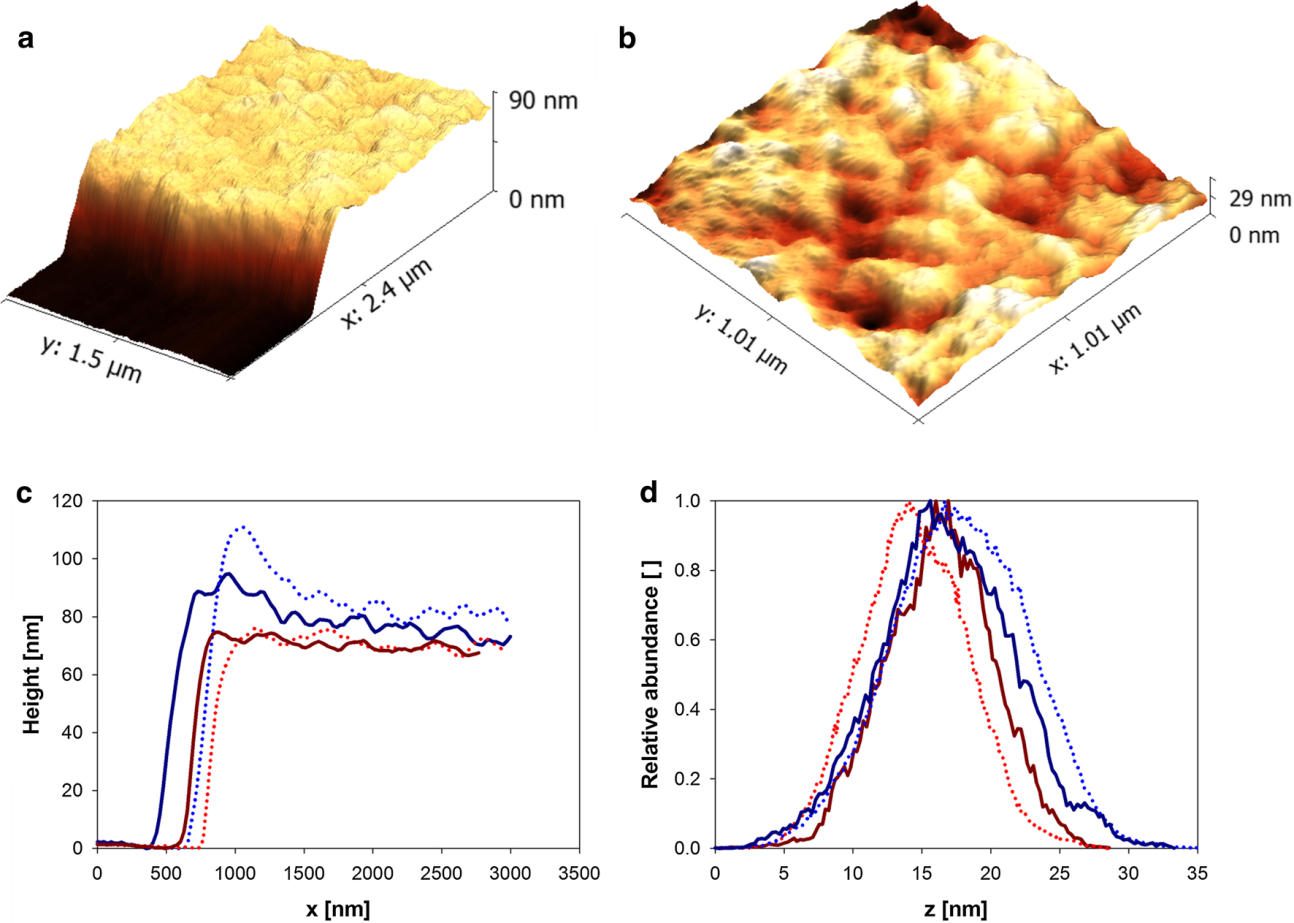
AFM imaging of SWO1 action on ATFC. **a** A three-dimensional representation of the experimental setup. ATFC of defined height is placed on a silicon wafer, which can be used as reference. **b** The ATFC surface is homogenous and nanoflat with a mean surface roughness below 5 nm. **c** ATFC substrates on a single silicon wafer (~1 cm^2^) were incubated in 50 mM sodium acetate buffer, pH 5.0, at 40 °C with mild agitation in a total reaction volume of 2 mL. Two exemplary height profiles from ATFC substrates after incubation with (*blue*) or without 0.4 µM SWO1 (*red*) after 24 h are shown. No significant changes induced by SWO1 incubation were found. Note that the edges of amorphous cellulose films were slightly deformed due to a cutting process prior to the addition of SWO1. Thus, only the surface with a certain distance (~1.0 µm) to the edge was analyzed. **d** Height distribution profiles of spots on the ATFC surface after incubation with (*blue*) or without SWO1 (*red*) using the same experimental conditions as stated above. A broadening of the peak, which would indicate degradation or swelling, is not visible. Analyzed areas were at least 1 µm^2^

Figure 6 shows the analysis of the amorphous cellulose treated with SWO1. The cellulose film provides a homogeneous and nanoflat surface for SWO1 to act upon (Fig. 6a, b). The overall surface roughness was below 5 nm. Height profiles were recorded from the surface at two representative regions of 1 µm^2^ area before and after treatment with SWO1. The results did not reveal changes in the surface topography as result of SWO1 action. Local effects of SWO1 on swelling or disruption of the cellulose surface, both of which would change the height, would have been clearly detectable with the method used (Fig. 6c, d). It appears, therefore, that on the amorphous cellulose used, SWO1 was inactive as a structure-loosening factor.

**Fig. 7:**
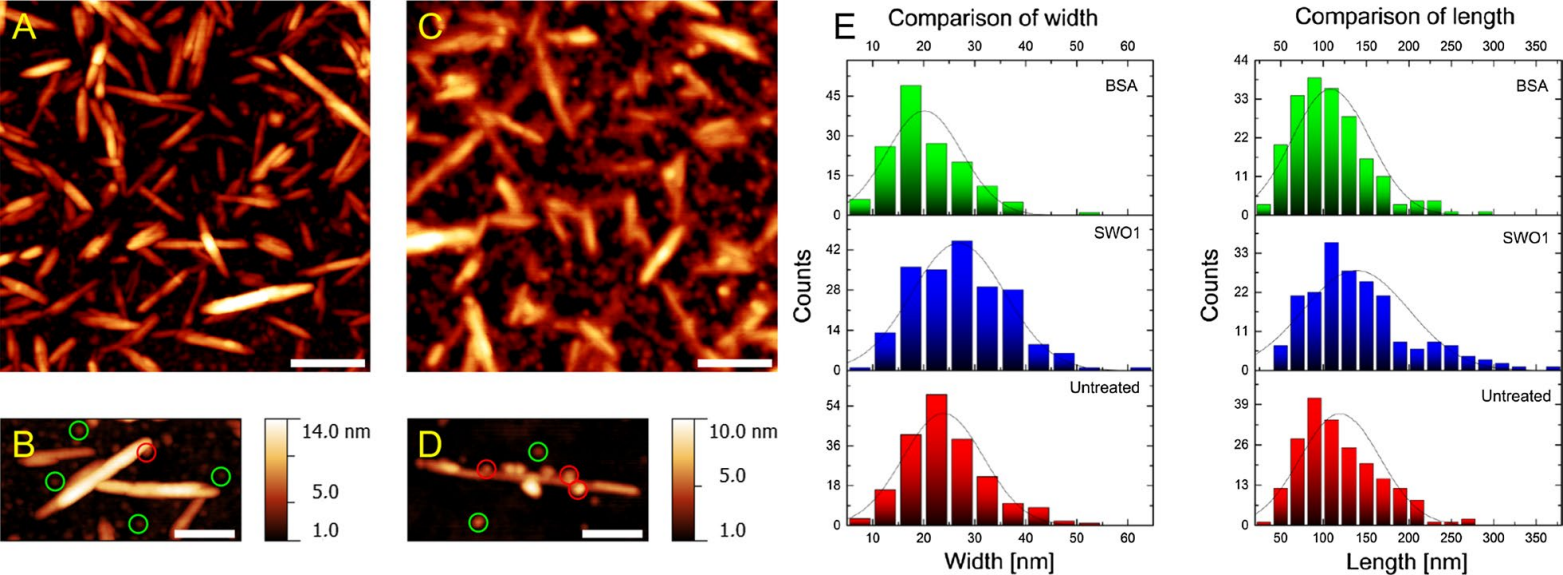
AFM imaging of SWO1 action on CNCs. CNCs on a single silicon wafer (~1 cm^2^) were incubated with either 0.4 µM BSA (**A**, **B**) or SWO1 (**C**, **D**) in 50 mM sodium acetate buffer, pH 5.0, at 40 °C with mild agitation. Incubation was done over 24 h in a total reaction volume of 2 mL. AFM imaging was done on dried silicon wafers at room temperature. No evidence for BSA- or SWO1-induced structural changes were found by visual examination. However, the presence of molecules attached to either CNCs or the silicon wafer can be observed (**A**–**D**). Most of the BSA molecules are positioned randomly on the silicon wafer (**A**). An exemplary amplified section is shown in **B**. Multiple BSA molecules are visible (*green circles*), and only one BSA molecule seems to be associated with a crystallite (*red circle*). Contrary, SWO1 showed a clear trend to become attached to CNCs (**C**). An exemplary amplified CNC confirmed that the ratio of molecules attached to crystals (*red circles*) and particles on the surface (*green circles*) has significantly increased (**D**). Note that for an easier viewing, not all BSA/SWO1 molecules are highlighted (**D**). **E** Statistical analysis of the size distribution showed an apparent increase in the width of CNCs upon incubation with SWO1. However, this effect is attributed to the size of the adsorbed protein and the presence of a hydration shell (see Fig. 8). *Scale bars* 100 nm

**Fig. 8:**
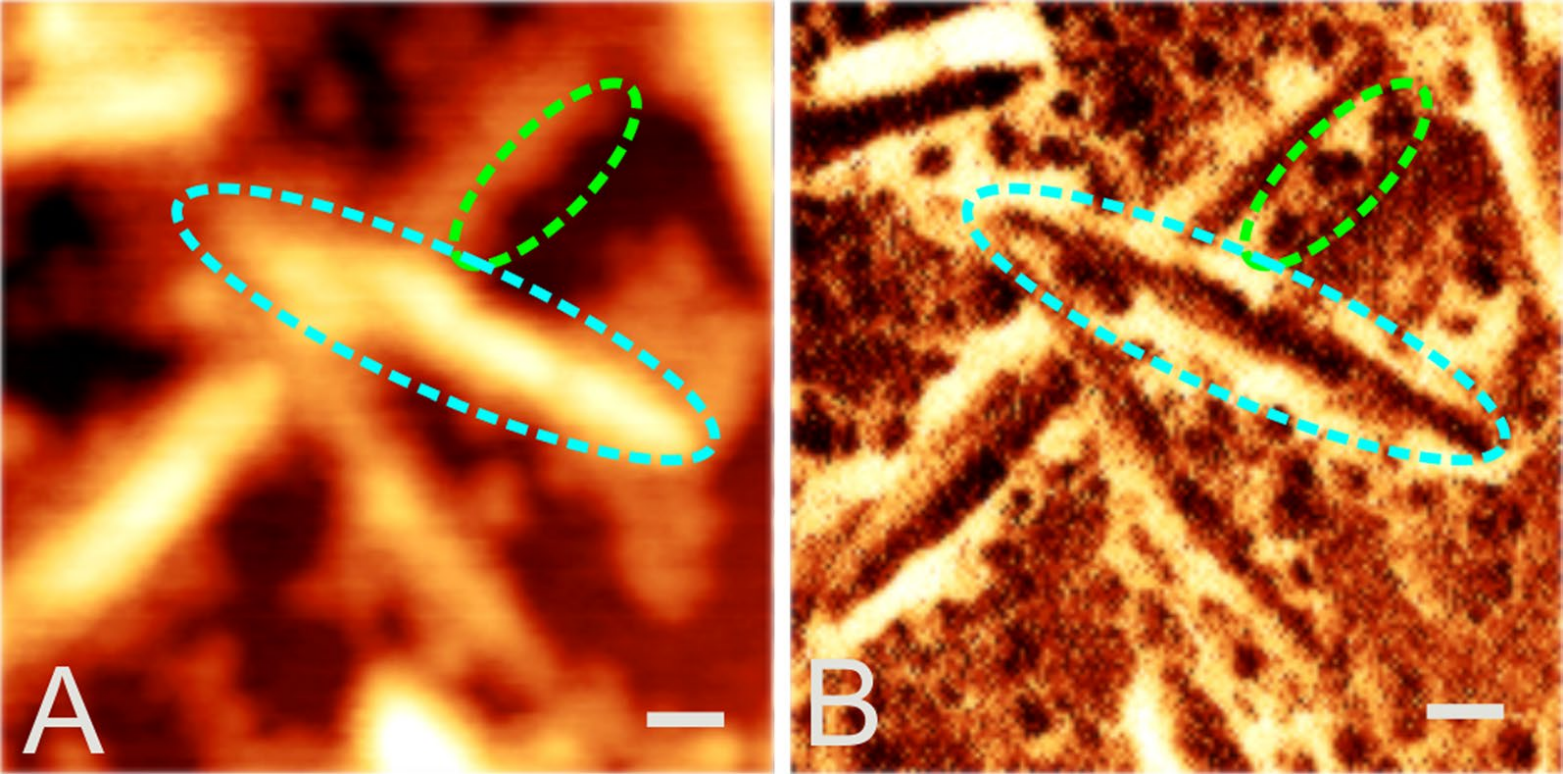
Details of SWO1 binding to CNCs revealed by AFM phase imaging. CNCs on a single silicon wafer (~1 cm^2^) were incubated with 0.4 µM SWO1 in 50 mM sodium acetate buffer, pH 5.0, at 40 °C with agitation. Incubation was done over 24 h in a total reaction volume of 2 mL. AFM imaging was done on dried silicon wafers at room temperature. **A** Recorded height images of CNCs are blurred, and structural features or proteins are not readily visible. **B** Phase imaging allowed the visualization of features like CNC-attached proteins (*green dashed ellipse*) covered by a hydration shell (bright layer enveloping CNCs). By comparison with the height image (*cyan dashed ellipse*), it is clear that the hydration shell is also, at least, partly responsible for the apparent broadening of the CNCs (Fig. 7E). The hydration shell is not present or significantly reduced upon incubation with BSA. *Scale bars* 30 nm

CNCs present a highly recalcitrant form of cellulose. From the herein applied method of their preparation [57, 63], the CNCs appeared as needle-like structures of about 100–200 nm length and about 3–70 nm width, as shown in Fig. 8. The CNCs were incubated with SWO1 and also with BSA as a control, and a representative area of each specimen (>1 µm^2^) was analyzed with AFM. In spite of extensive data analysis that involved the characterization of numerous CNCs in each image (*N* ≥ 20) at different levels of their structure, significant changes in the substrate were recognized neither in the SWO1 incubation nor in the BSA control. To examine the samples in more detail, they were washed after 24 h of incubation, dried and again analyzed with AFM. Results in Fig. 7A, B served to localize single BSA molecules, showing that they were attached mostly to the surface of the silicon wafer, apparently in a random fashion, and only occasionally to the CNCs. The situation was notably different when SWO1 was used, as shown in panels C and D of Fig. 7. Despite unspecific binding to the wafer surface to some degree, SWO1 showed the clear trend to become enriched around the CNCs (panel C). Imaging of single CNCs at a resolution down to single protein particles revealed multiple sphere-like SWO1 molecules bound at both sides of the cellulose rod (panel D). Specific adsorption of SWO1 to CNCs is, therefore, suggested to surpass a mere adhesion of the protein to the hydrophobic surface of the wafer. Analysis of the distribution of height and width in multiple CNCs after the incubation and comparison of the result with the corresponding distribution of the untreated sample reveal that BSA really had no effect whatsoever on the size properties of the cellulosic substrate (Fig. 7E). SWO1, by contrast, caused the width distribution to shift by about 3 nm to an elevated mean value (Fig. 7E). Adsorption of SWO1 along the sides of the nanocrystals is likely to have caused this effect.

Interesting observation from these AFM analyses was that despite having a similar apparent mass like BSA (~66 kDa), SWO1 particles appeared distinctly larger (10–30 nm diameter) in the images than BSA particles, as recognized clearly when comparing panels B and D in Fig. 7. We think that the high glycosylation of SWO1 and the consequently pronounced hydration of the protein could explain the effect. Features of the cellulose nanocrystals recorded from the SWO1 experiment appear strongly blurred in comparison to the BSA experiment, which is most likely related to hydration. Figure 8 illustrates the effect in more detail, comparing height (panel A) and phase (panel B) images from the experiment with SWO1. Phase imaging depicts the dissipative interaction energy density and allows a clear distinction between materials with different characteristics (e.g., CNCs and enzymes). By comparison with the height data, the phase image reveals the presence of multiple structural features, which are convoluted and blurred in the height image. First, CNCs appear to be thinner in phase imaging, and second, CNCs are surrounded by a bright layer with a unique phase signal, which represents most likely a hydration shell. In addition, SWO1 molecules, either free or attached to CNCs, embedded in the hydration shell can be observed, which are not readily visible in the height image (Fig. 8). The hydration shell is contributing to the apparent broadening of the CNCs and is not present or significantly reduced upon incubation with BSA.

### Synergy between SWO1 and *T. reesei* cellulases in releasing soluble sugars from different lignocellulosic substrates

**Fig. 9:**
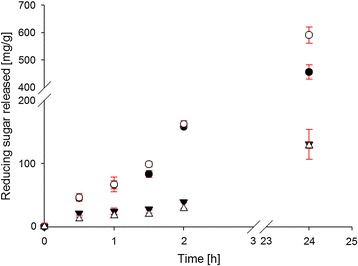
Effect of SWO1 pretreatment on the enzymatic hydrolysis of cellulosic substrates. The substrates used were Avicel PH-101 (○/●) and CNCs (Δ/▼). The substrate concentration was 1 mg/mL. All reactions were done in 50 mM sodium acetate buffer, pH 5.0, at 40 °C with shaking (500 rpm) in a total reaction volume of 1.5 mL. Prior to the addition of cellulase, the substrate preparation was incubated with 0.4 µM SWO1 (●/▼) or BSA (○/Δ) for 24 h. *T. reesei* cellulase and β-glucosidase were then added in a small volume (60 µL) to a final enzyme loading of 20 µg/mg substrate and 4 µg/mg substrate, respectively. The mixture was incubated for another 24 h using the same conditions as stated above. The liberated glucose was measured with an enzymatic assay. *Error bars* show SD from four independent experiments

Ability of SWO1 to boost the hydrolysis of different lignocellulosic substrates by the complete *T. reesei* cellulase system was analyzed. Figure 9 shows time courses of reducing sugar release from Avicel PH-101 and CNCs under conditions in which the celluloses were pre-incubated with SWO1 or BSA for 24 h and cellulases were then added to initiate the hydrolysis. The results are clear in showing that SWO1 did not enhance the substrate conversion. We also examined the effect of SWO1 on the hydrolysis of filter paper but found none. Note: using light microscopy, we further analyzed if incubation with SWO1 alone caused disintegration of the fibrous filter paper material. This did not occur. Wheat straw, that had been pretreated by steam explosion, and birchwood xylan were also tested as substrates of enzymatic hydrolysis in the absence and presence of SWO1. Again, there was no boosting effect by SWO1 within the limits of experimental error, and the SWO1 lacked sugar-releasing activity on its own.

**Fig. 10:**
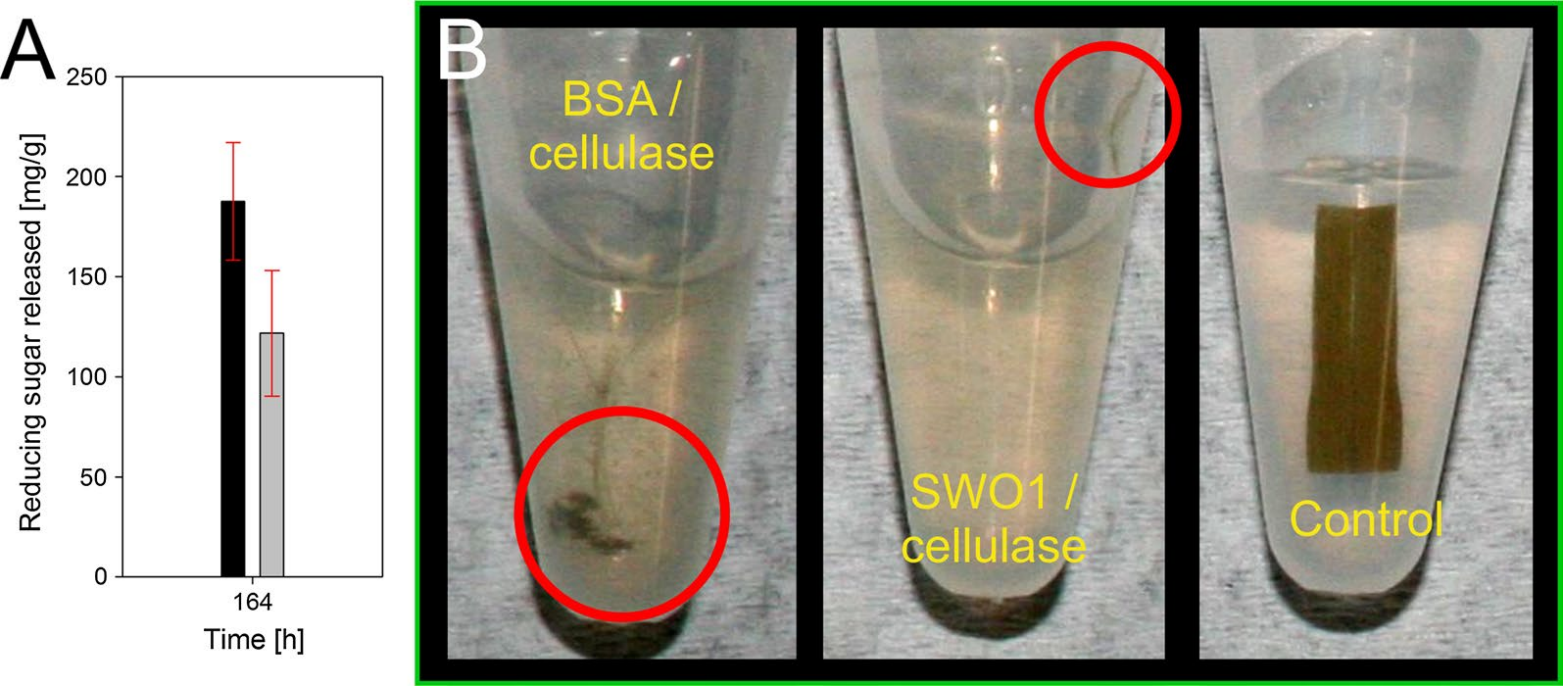
Effect of SWO1 supplementation on the enzymatic hydrolysis of cock’s-foot grass. **A** Reactions were done in 50 mM sodium acetate buffer, pH 5.0, at 40 °C with shaking (500 rpm) in a total reaction volume of 1 mL over 164 h. Substrate concentration was 5.0 mg/mL, and cellulase was added to a final protein loading of 2 µg/mg substrate. SWO1 was present at 0.02 µM (*black bar*), and the reference experiment used an equimolar amount of BSA (*grey bar*) instead of SWO1. The amount of reducing sugars released was measured colorimetrically with the 3,5-DNS assay calibrated against glucose. *Error bars* were estimated from two independent experiments. **B** By comparison with the BSA-containing control reaction (*left panel*), cock’s-foot grass appeared to be more completely degraded in the presence of SWO1 (*central panel*) after 164 h. The remaining substrate parts are highlighted for an easier viewing. The untreated material is shown as reference (*right panel*)

Finally, we examined a lignocellulose substrate, which, except for drying, had not been pretreated at all. A sample of cock’s-foot grass (*Dactylis glomerata*) was used. Figure 10 compares the sugar release and also the visual appearance of the substrate after incubation with cellulases in the presence and absence of SWO1. Compared to the BSA control, the reaction containing the SWO1 showed a 1.5-fold improved sugar formation and appeared more completely degraded. The untreated material is shown for reference.

## Discussion

### Preparation of recombinant native-like SWO1 via homologous expression in *T. reesei*

Despite different approaches tried, as shown in Table 1, preparation of the *T. reesei* SWO1 in a recombinant form is currently not well established. Most studies seem to agree, at least implicitly, that recombinant production of SWO1 is highly problematical due to the very low protein titers formed in different host organisms. There is, however, good evidence already from the seminal discovery of Saloheimo et al. [18] that the native SWO1 is strongly post-translationally modified. The protein is glycosylated [37, 43, 48] and its carbohydrate-binding module likely involves multiple disulfide bonds [39]. Since it is not known in which way the post-translational modifications affect the function of SWO1, we considered it crucial to prepare the protein in its native host and additionally avoided the use of purification tags, which also bear the risk of affecting the function in an unpredictable fashion. The native-like SWO1 so obtained was purified to apparent homogeneity from *T. reesei* culture supernatant. The isolated protein was shown to be heavily glycosylated. From analysis of the apparent molecular mass before and after treatment with endo-*N*-glycosylase, we concluded that *N*-glycans constituted only a small portion of the total protein-linked glycans present. SWO1, thus, appeared to be *O*-glycosylated in substantial amount. From its CD spectrum, the protein seemed to be properly folded. Its functional characterization was considered to be of a general interest as it could provide basic evidence to advance the current debate about a possible role of SWO1 as C_1_ factor of enzymatic lignocellulose degradation. Interpretation of any C_1_-like activity that a certain recombinant form of SWO1 may show, as such or relative to another form produced differently, hinges essentially on an assessment of the nativeness of the protein used. This, in turn, requires knowledge about the behavior of the canonical (native) form of SWO1.

### Characteristics of function of the native-like SWO1

Although SWO1 binds to crystalline cellulose, as we have shown here in equilibrium adsorption studies and for the first time at single-molecule resolution by AFM, it has a much higher specificity for binding to xylan as compared to cellulose. Differences in binding specificity are not only a consequence of a different binding affinity, which is higher for xylan, but also reflect a substantially larger binding capacity of SWO1 on xylan than cellulose (Avicel PH-101). Adsorption experiments with lignin demonstrate the ability of SWO1 to bind also to the non-carbohydrate residue of lignocellulose with relatively high specificity. Interestingly, a recombinant swollenin from *A. fumigatus* produced in *A. oryzae* was reported not to bind to xylan [34]. Contrarily, most bacterial expansins are reported to bind on xylan [21, 26, 54, 64, 65].

By employing assays and analytical techniques able to capture even subtle effects of an SWO1-caused structural disintegration of amorphous and crystalline cellulose, we gathered a considerable body of evidence coherent in the overall suggestion that the native-like SWO1 was essentially inactive as an “amorphogenesis” factor on pure celluloses. Neither did SWO1 loosen or roughen up the cellulose surface [18, 34, 37] nor did it cause swelling of the cellulose material [18, 28, 37]. But also the opposite effect, that SWO1 smoothens an otherwise rough cellulose surface [41], was not observed. However, a noticeable effect from the incubation of CNCs with SWO1 was that after drying, there remained an apparent hydration shell, which surrounded the absorbed SWO1 molecules and the CNCs. It is known from molecular dynamics simulations [66] and low-field nuclear magnetic resonance studies [67, 68] that the surface hydration is a key parameter in enzymatic cellulose hydrolysis, affecting both the enzyme adsorption and the conversion. In general, a higher degree of hydration is beneficial; however, the availability of “free” water [67, 68] and the water activity [69] seem to be particularly important. A protein retaining its hydration shell even after drying is quite unlikely to increase the amount of readily available water at the cellulose surface, for activity of the hydrolytic enzymes or structural changes in the substrate requiring penetration of water. Thus, the evidence from the AFM imaging also suggests that SWO1 is probably not an “amorphogenesis” factor on pure cellulose. However, the use of a protein with a substantial hydration layer could be of interest when using high solid loadings. In recent studies, it was hypothesized that constrained low-entropy water significantly contributes to the biomass recalcitrance in polymer suspensions (≥10 % dry solid, *w/w*) [68]. A proposed mechanism includes the release of energetically unfavorable water, so revealing hydrophobic spots on the substrate surface and, thus, facilitating unproductive binding of the cellulases. In addition, the formation of steric hindrances due to polymer–polymer junction zones might be possible as a result [68]. It is conceivable that SWO1 with its substantial hydration layer could cover these energetically unfavorable hydrophobic spots and thereby affect the conversion yield positively. However, it is worth noticing that even BSA is reported to exert a similar function in lignocellulose pretreatment by reducing the unproductive adsorption of cellulases to lignin [70]. However, we have to emphasize strongly that those images were recorded under non-physiological conditions. These findings are clearly at variance, and appear difficult to reconcile, with a number of recent papers and also the original work of Saloheimo et al. [18], reporting a cellulose structure-altering activity of the respective SWO1 preparation used. The question of how much of the difference in the findings can be attributed to the varying SWO1 preparations used is difficult to answer. Significant variations in the experimental conditions used are noted. However, even experiments with a comparable setup gave widely differing results. For instance, using Avicel PH-101, which is a commonly applied and well-characterized model substrate for crystalline cellulose [58, 71, 72], a number of studies reported a reduction in particle size after incubation with SWO1 [34, 37, 38, 40]. Furthermore, three studies using Avicel PH-101 from the same manufacturer, comparable enzyme loadings, incubation time and temperature also tried to quantify the particle size reduction [34, 37, 40] (see Table 1). Despite high similarity of the experimental setup used, the size reduction varied between ~50 % [34], ~25 % [37] and even nil in one study [40]. Thus, we think that SWO1 is probably a prime source of variability in the different studies.

Georgelis et al. [20] examined several expansin-like proteins from different microorganisms, including *Aspergillus niger*. They found all proteins to be active in a cell wall extension assay, whereas none of them showed synergy with individual *T. reesei* cellulases or the complete enzyme complex hydrolyzing filter paper. The picture emerging from several studies of expansin synergy with cellulases is that expansin exhibits highest effectiveness when lignocellulosic feedstocks, not pure celluloses, are used as the substrates [44, 54, 73]. This notion is in agreement with our finding that SWO1 prefers to bind to xylan and that synergy with cellulases was detectable on an untreated lignocellulosic substrate. Gourlay et al. [44] reported large factors of synergy between individual xylanases and SWO1 in the release of xylose from steam-pretreated corn stover. Summing up, these findings fit quite well together and already suggest a potential structural target for synergistic interplay of SWO1 with cellulases and hemicellulases, respectively. Moreover, in earlier studies, xylan and xylooligomers [68, 74, 75] were recognized as potent inhibitors for cellulases. Thus, understanding and overcoming inhibition caused by xylans and xylooligomers eventually would be highly interesting from a scientific and applied point of view. However, we did not observe synergy between SWO1 and *T. viride* β-xylanase M1 (data not shown) in the conversion of xylan into reducing sugars. Overall, the effectiveness of SWO1 in acting in synergy with cellulases and hemicellulases deserves further systematic investigation. Ultimately, our results suggest that SWO1 is not a C_1_ factor of degradation of pure cellulose. Still, there is a possibility that unknown proteins or co-factors (e.g., metals) are necessary to fully unlock the potential of SWO1 in that function. Although, according to our knowledge, there is no evidence in the literature for additional proteins or factors required to promote the activity of SWO1 or expansins in general, this possibility might inspire promising future investigations.

## Conclusions

In summary, some basic biochemical characteristics of the native SWO1 were presented. The protein is strongly glycosylated. O-glycosylation appeared to predominate over N-glycosylation. Results of CD spectroscopic characterization agree with evidence from molecular modeling, suggesting a folded protein with a high relative content of β-strands. Although SWO1 binds to crystalline cellulose, its adsorption to xylan is much stronger. A role of isolated SWO1 as a factor of amorphogenesis of pure cellulose was not supported. According to the classical C_1_–C_x_ postulate, SWO1 is not a C_1_ factor of degradation of the pure cellulosic substrates examined herein, neither in regard to affecting their morphology on adsorption, nor to acting in synergy with the cellulases in their hydrolysis. However, the release of sugar from barley β-glucan and cellotetraose might reflect a weak intrinsic hydrolase activity of SWO1. Synergy with *T. reesei* cellulases strongly depended on the substrate used. While absent with pure celluloses, a slight beneficial effect of SWO1 on soluble sugar release from untreated biomass sample with intact plant cell walls was observed. This might be relevant, with certain (e.g., mildly pretreated) lignocellulosic substrates, and even exploitable if the effect is preserved at increased substrate loadings.

## Methods

### Enzymes and substrates

Complete *T. reesei* cellulase was from fungal culture (strain SVG17) on wheat straw. *Tr*CBH I was purified from the cellulase mixture using a reported ion exchange protocol [76]. Serum albumin fraction V (BSA) was bought from Roth (Karlsruhe, Germany), β-glucosidase from *Aspergillus niger* and β-xylanase M1 from *T. viride* were obtained from Megazyme International (Wicklow, Ireland). Avicel PH-101 and lignin (alkali, low sulfonate content) were obtained from Sigma-Aldrich (St. Louis, MO, USA), barley β-glucan (high viscosity > 100 cST) from Megazyme International (Wicklow, Ireland), birch xylan from Roth (Karlsruhe, Germany), cellotetraose and 4-methylumbelliferyl-β-d-cellobioside from Carbosynth (Compton, UK). CNC was prepared from Whatman^®^ qualitative filter paper (Sigma-Aldrich, St. Louis, MO, USA) using H_2_SO_4_ according to Lu et al. [63]. Amorphous thin film cellulose (ATFC) was prepared from trimethylsilyl cellulose by a reported procedure [57].

### Construction of a *T. reesei* expression strain for SWO1 production, and culture conditions used

*T. reesei* Δ*xyr1* was used as the recipient strain for the *swo1* expression plasmid and maintained on potato dextrose agar at 28 °C. The strain is deleted in the major cellulase and xylanase regulator *xyr1* and derived from strain QM9414 (ATCC 26921). Fermentations were carried out in Biostat^®^ A Plus bioreactors (Sartorius, Göttingen, Germany) in a 2-L working volume. One liter of fermenter medium comprised 4.6 g (NH_4_)_2_SO_4_, 3 g KH_2_PO_4_, 0.3 g MgSO_4_·7H_2_O, 0.4 g CaCl_2_, 20 mL of 50× trace elements solution (250 mg/L FeSO_4_·7H_2_O, 80 mg/L MnSO_4_·H_2_O, 70 mg/L ZnSO_4_·7H_2_O, 100 mg/L CoCl_2_·2H_2_O), 0.5 mL Tween 80 and 50 g d-glucose. The fermenter was inoculated with a preculture. Therefore, about 10^6^ spores/mL were added to 250 mL minimal medium [49] in a 1-L Erlenmeyer flask and grown for about 24 h at 28 °C in a rotary shaker at 250 rpm. Fermentation conditions were 28 °C, 500 rpm, an air flow rate of 2–3 L/min and pH 5.0 adjusted with 1 M NH_4_OH or 1 M HCl. Supernatants were separated from fungal biomass by centrifugation for 20 min at 4 °C and 4200 rpm followed by filtration of the supernatants through a Miracloth sheet (Calbiochem, San Diego, CA, USA). Samples were stored at −20 °C prior to purification.

### Construction of *swo1*-expressing *T. reesei* strains

The *swo1* coding region (XP_006969225.1) including 575 bp of its terminator region was PCR-amplified with primers infuse_swo1_fw (5′-caacttctctcatcgatgaactgttagacgggatggc-3′) and infuse_swo1_rv (5′-tgcaggtcgacatcgatgcgtgcctgtgtatcaattg-3′) from genomic DNA of *T. reesei* QM6a (ATCC13631) and cloned into the ClaI-digested pLH_hph_Pcdna1 expression plasmid using the InFusion^®^ HD Cloning Kit (Clontech Laboratories, Inc., Mountain View, CA, USA). This *swo1* expression plasmid (p_swo1oe) contains the hygromycin B phosphotransferase (*hph*) expression cassette as fungal selection marker and 930 bp of the *T. reesei cdna1* promoter region [49] to drive *swo1* expression. DNA fragments were purified using the QIAquick gel extraction kit (QIAGEN GmbH, Hilden, Germany). The circular p_swo1oe was used to transform *T. reesei* QM9414Δ*xyr1* via electroporation. Conidia of a fully sporulated PDA Petri dish (Difco, Detroit, MI, USA) were harvested, filtered through glass wool and inoculated in 100 mL of YPD (10 g/L yeast extract, 20 g/L peptone) +2 % d-glucose followed by incubation in a rotary shaker for 4 h at 30 °C and 300 rpm. Then, the conidia were pelleted, washed three times with cold 1.1 M d-sorbitol (Alfa Aesar GmbH & Co KG, Karlsruhe, Germany), and resuspended in 300 µL of cold 1.1 M d-sorbitol. Seventy-five-microliter aliquots were mixed with 10–15 µL (10–30 µg) of p_swo1oe and electroporated at 1.8 kV using 0.1-cm cuvettes in a MicroPulser (Biorad, Hercules, CA, USA). Thereafter, cells were recovered in a premixed solution of 400 µLöö 1.1 M d-sorbitol + 125 µL YPD and incubated in a Thermomixer (Eppendorf, Hamburg, Germany) for 1 h at 28 °C and 800 rpm before plated on selection medium (PDA + 100 mg/L hygromycin B (Carl Roth + Co KG, Karlsruhe, Germany). Transformants were purified by single spore isolations on selection medium containing 0.1 % *v/v* Triton X-100. From the transformants, genomic DNA was extracted, and the presence of the *swo1* expression cassette was verified by diagnostic PCR using the primer swo1_conf_for (5′-GCCGGCTTCAAAACACACAG-3′) and swo1_conf_rev (5′-GTTGTGTGGAATTGTGAGCGG-3′) resulting in a 2.2-kb fragment in positive transformants. Expression of SWO1 in positive strains was examined using SDS-PAGE. The culture supernatant was analyzed, and the strain designated as RJ_SWO1 was used for further studies.

### Purification of SWO1 from *T. reesei* culture supernatant

The supernatant was thawed and centrifuged (5 min, 5000 rpm, 4 °C). About 50 mL of supernatant (~2 mg total protein) was mixed with 50 mL of sodium acetate buffer (50 mM; pH 5.0), and Avicel PH-101 (2 g) was added. The suspension was stirred for 2 h at room temperature. Avicel PH-101 was first separated from the supernatant by sedimentation and then washed three times with 50 mL of the same sodium acetate buffer. The Avicel PH-101 was recovered by centrifugation (5 min, 5000 rpm, 4 °C) and then packed under gravity into a disposable 10-mL polypropylene gravity flow column (Thermo Fisher Scientific, Waltham, MA, USA). The column was washed twice in each case with adsorption buffer and doubly distilled H_2_O to remove non-specifically adsorbed protein. SWO1 was eluted with 1 % triethylamine (TEA) in doubly distilled H_2_O and collected in an excess of gently mixed adsorption buffer. The eluted fraction was concentrated using ultrafiltration concentrator tubes (Vivaspin^®^6, MWCO 10 kD) from Sartorius (Goettingen, Germany). SDS-PAGE with Coomassie Brilliant Blue staining showed a single, slightly diffusive protein band with the expected apparent molecular mass.

Note that a minor fraction (≤20 %) of partly purified SWO1 was already eluted during the washing step with water. We conducted preliminary adsorption experiments using Avicel PH-101 as adsorbent with both SWO1 fractions. The SWO1 eluted with 1 % TEA showed a slightly higher affinity to Avicel PH-101, however, within the range of experimental error (data not shown). To avoid ambiguities, we only used the SWO1 fraction eluted with TEA in all experiments reported from this study.

The protein concentration of solutions of purified SWO1 was determined by intrinsic UV-absorption on a DeNovix DS-11 spectrophotometer (DeNovix Inc., Wilmington, DE, USA). The molar extinction coefficient of SWO1 was determined from the protein sequence from UniProt using ProtParam (ε_SWO1 (Q9P8D0) = 88,655 M^−1^ cm^−1^). The purified and concentrated SWO1 was stored at 4 °C.

### Deglycosylation of SWO1

Deglycosylation of SWO1 was performed according to a standard Endo H protocol (New England Biolabs, Frankfurt, Germany). The purified protein (10 µg) was mixed with 10× denaturation buffer (5 % SDS, 400 mM DTT) up to 40 µL of total volume and incubated at 95 °C for 10 min. Subsequently, one-tenth volume of 10× G7 reaction buffer (500 mM sodium phosphate buffer, pH 7.5) was added. The mixture was incubated with 300 U Endo H at 37 °C for 90 min. Reaction was stopped by heating for 10 min to 95 °C. Deglycosylated samples and untreated controls were analyzed by SDS-PAGE (NuPAGE^®^ Bis–Tris 4–12 %) with glycostaining and subsequent Coomassie Brilliant Blue staining (Thermo Fisher Scientific, Waltham, MA, USA). Glycostaining was done with the Pro-Q Emerald 300 glycostain kit (Invitrogen, Carlsbad, CA) according the manufacturer’s protocol.

### Circular dichroism and modeling of reference data

Circular dichroism spectra were acquired on a Jasco J-175 spectropolarimeter (Jasco Analytical Instruments, Groß-Umstadt, Germany) using a 10-mm cylindrical quartz cell. SWO1 was used at a concentration of 0.1 mg/mL in 50 mM sodium acetate buffer, pH 5.0, at room temperature. The baseline of the spectra was obtained from pure buffer. The standard parameters for protein evaluation were chosen with a sensitivity of 100 mdeg, a start wavelength of 250–320 nm, an end wavelength of 190–250 nm and a data pitch of 1 nm. For good data quality, a slow scanning mode with a continuous scanning speed of 10 nm/min was chosen. The combined spectra were evaluated online with DichroWeb, which calculated the secondary structure elements of SWO1.

To obtain reference date for SWO1, the automated protein structure homology-modeling server Phyre2 was used to predict the protein structure of SWO1 [50]. The sequence of SWO1 from UniProt (ID: Q9P8D0) was used except for the amino acids 1–18, which are predicted to be cleaved off after secretion. The obtained protein model was used to calculate the percentage of secondary structure elements. A second set of reference data was obtained using the sequence-based JNet algorithm (JPred4) (http://www.compbio.dundee.ac.uk/jpred4/index.html) to calculate the percentage of secondary structure elements as described elsewhere [51].

### Characterization of SWO1 binding affinity

All adsorption isotherm measurements were carried out in 1.5-mL Eppendorf tubes containing serial dilutions of SWO1 (0.2–25 µM) mixed with an equal volume of an aqueous suspension of substrate (Avicel PH-101, birchwood xylan and lignin) to a final concentration of 1 mg/mL in 50 mM sodium acetate buffer (pH 5.0) in a total reaction volume of 200 µL. All adsorption experiments were conducted in triplicates at 25 °C with orbital shaking (500 rpm) over 2 h using an Eppendorf Thermomixer comfort (Eppendorf AG, Hamburg, Germany). An incubation time of 2 h was sufficient to reach adsorption equilibrium according to previously published articles [37] and preliminary experiments on Avicel PH-101 (data not shown). The samples were then centrifuged (5 min, 13,000 rpm, 25 °C) to remove solids. The clear supernatant was collected, and protein concentration was determined by BCA (Thermo Fisher Scientific, Waltham, MA, USA) (Avicel PH-101) and Roti-Nanoquant assay (Carl Roth + Co KG, Karlsruhe, Germany) (xylan and lignin). *Tr*CBH I was used as standard. The equilibrium association constants (*K*_d_) were determined by nonlinear regression of bound versus free protein concentrations to Langmuir model as described previously. A control showed that unspecific binding of SWO1 to the reaction tubes was negligible.

### Activity of SWO1 on crystalline cellulose substrates

SWO1 was incubated with Avicel PH-101 or CNC in 50 mM sodium acetate buffer, pH 5.0, in a total reaction volume of 500 µL at 40 °C and 500 rpm using an Eppendorf Thermomixer comfort (Eppendorf AG, Hamburg, Germany). Experiments were done in four replicates, and the substrate concentration was 1.0 mg/mL (Avicel PH-101 or CNC). Samples were incubated with either 0.4 µM SWO1 or an equimolar amount BSA. Samples were taken after 12 and 24 h, respectively. About 100 µL of the supernatant was withdrawn and heated to 95 °C for 10 min to stop the reaction. Subsequently, the samples were centrifuged (13,000 rpm, 1 min, 25 °C), and β-glucosidase was added to a final concentration of 2 µg/mL to the cleared supernatant. The reaction mixture was incubated for 1 h at 37 °C to convert of released cello-oligosaccharides to glucose. Finally, the amount of released glucose in the supernatant was assayed colorimetrically with glucose oxidase and peroxidase as described in earlier works [12].

### Hydrolysis of β-glucan and cellotetraose by SWO1

Hydrolysis of soluble glucans was studied at 1 mg/mL (β-glucan) or 0.5 mg/mL (cellotetraose), respectively. All experiments were carried out as duplicates in 50 mM sodium acetate buffer, pH 5.0, in a total reaction volume of 400 µL at 40 °C and 500 rpm. The reaction was started with the addition of SWO1 or an equimolar amount of BSA. The final enzyme concentration was 0.2 (β-glucan) or 0.5 µM (cellotetraose), respectively. The reaction was stopped after 24 h by adding an equal volume of 100 mM NaOH. Precipitated material was removed by centrifugation (3 min, 13,000 rpm, 25 °C). The cleared supernatant was analyzed with high-performance anion-exchange chromatography coupled to pulsed-amperometric detection (HPAEC-PAD) (Dionex BioLC, Thermo Fisher Scientific, Waltham, MA) as described elsewhere [57]. Identity and amount of released sugars were assayed using authentic standards.

### Enzymatic hydrolysis of crystalline cellulose pretreated with SWO1

The impact of SWO1 supplementation on the hydrolysis of a typical fungal cellulase set was assayed in 50 mM sodium acetate buffer, pH 5.0 at 40 °C and 500 rpm in an Eppendorf Thermomixer comfort (Eppendorf AG, Hamburg, Germany). Substrate concentration was 1.0 mg/mL of cellulose (Avicel PH-101 or CNC) in a total reaction volume of 1.5 mL. Reactions were repeated four times. Pretreatment was done over 24 h with 0.4 µM SWO1 or BSA as reference. Afterward, *T. reesei* cellulase and β-glucosidase were added in a small volume (60 µL) to a final enzyme loading of 20 µg and 4 µg/mg substrate, respectively.

Sampling was performed at suitable time points. In brief, 150 μL of the well-mixed supernatant was withdrawn and heated to 95 °C for 10 min to stop the reaction. Subsequently, the samples were centrifuged (13,000 rpm, 1 min, 25 °C), and the amount of released glucose in the supernatant was assayed colorimetrically with glucose oxidase and peroxidase as described above.

### Enzymatic hydrolysis of lingocellulosic material with supplementation of SWO1

Hydrolysis experiments with *Dactylis glomerata* grass were conducted in duplicates in a parallel assay. Fifty mM sodium acetate buffer, pH 5.0, was used in a total volume of 1 mL at 40 °C. The samples were shaken at 500 rpm in an Eppendorf Thermomixer comfort (Eppendorf AG, Hamburg, Germany). Prior to hydrolysis experiments, *Dactylis glomerata* grass was dried at 80 °C overnight. Substrate concentration was 5.0 mg/mL, and cellulase was added to a final protein loading of 2 µg/mg substrate. SWO1 was present at 0.02 µM, and the reference experiment used an equimolar amount of BSA instead of SWO1. Sampling was performed at suitable times. About 100 µL of the well-mixed supernatant was withdrawn and mixed with 100 μL of 100 mM NaOH to stop the reaction. Subsequently, the samples were centrifuged (13,000 rpm, 1 min, 25 °C), and the amount of released sugars in the cleared supernatant was assayed colorimetrically with the 3,5-dinitrosalicylic acid-based assay calibrated against glucose [77].

### Atomic force microscopy (AFM) imaging of SWO1 activity

Investigations of the incubated samples were carried out on a FastScan Bio AFM (Bruker AXS, CA, USA), operated by a NanoScope V controller and FastScan A cantilevers (Bruker AXS, CA, USA) at room temperature. Setpoints, scan rates and controlling parameters were chosen carefully to ensure lowest possible energy dissipation to the sample and to exclude tip-driven artifacts.

Prior to AFM investigation, fully amorphous (ATFC) and highly crystalline cellulose (CNC) preparations were spin-casted on silicon wafers as described previously. Experiments were conducted as duplicates at 40 °C in a water bath with mild agitation. A single silicon wafer covered with cellulosic material was used as the substrate in a total reaction volume of 2 mL of 50 mM sodium acetate buffer (pH 5.0). Cellulosic material was equilibrated in buffer for 30 min prior to the addition of enzyme. The respective enzyme, SWO1 or BSA (only CNC) as negative control, was added to a final concentration of 0.4 µM. The reaction was conducted over 24 h and stopped by removing the silica wafers from the reaction mixture and rinsing them with 15 mL doubly distilled H_2_O to remove salt crystals.

Afterward, CNC-coated silica wafers were dried for 24 h up to 48 h at room temperature prior to AFM investigations. Silica wafers coated with ATFC were stored at 4 °C in doubly distilled H_2_O and examined in a laboratory-built liquid cell. The storage time did not exceed 6 h.

AFM image processing and analysis was performed using NanoScope Analysis 1.50 (Build R2.103555, Bruker AXS, CA, USA) and Gwyddion 2.31 (Released 2013-02-01, http://gwyddion.net/).

### Wide-angle x-ray scattering (WAXS)

Wide-angle x-ray scattering analysis (WAXS) was carried out on a Siemens D 5005 diffractometer (Siemens, Berlin, Germany) using CuKα (0.154 nm) radiation at 40 kV and 40 mA. 10 mg/mL Avicel PH-101 was incubated with 0.01 µM SWO1 for 72 h in a shaking water bath (GFL 1083) at 50 rpm. As a reference, Avicel PH-101 without SWO1 incubation was used. The probes were dried at 60 °C overnight, and were put on a zero-diffraction silicon crystal holder (Bruker AXS, CA, USA). All samples were characterized in locked coupled Θ/2Θ mode from 10° to 60° (Θ/2Θ) with an angle increment of 0.05° in 6 s. Data analysis was performed using Origin 9.0 (OriginLab Corp., Northampton, MA, USA).

## Abbreviations

ATFC: amorphous thin film cellulose
BSA: bovine serum albumin
CAE: congo red adsorption enhancement
CBH: cellobiohydrolase
CBM: carbohydrate-binding module
CNC: cellulose nanocrystals
EG: endoglucanase
ESEM: environmental scanning electron microscope
Fn-III: fibronectin-III
HPAEC-PAD: high performance anion exchange chromatography coupled to pulsed-amperometric detection
SD: standard deviation
SEM: scanning electron microscope
TEA: triethylamine
WAXS: wide-angle x-ray scattering analysis
